# Contrasting Responses of Rhizosphere Bacteria, Fungi and Arbuscular Mycorrhizal Fungi Along an Elevational Gradient in a Temperate Montane Forest of China

**DOI:** 10.3389/fmicb.2020.02042

**Published:** 2020-08-20

**Authors:** Yaoxin Guo, Chengjie Ren, Junjie Yi, Russell Doughty, Fazhu Zhao

**Affiliations:** ^1^Key Laboratory of Resource Biology and Biotechnology in Western China (Ministry of Education), Northwest University, Xi’an, China; ^2^College of Agronomy, Northwest A&F University, Yangling, China; ^3^Department of Microbiology and Plant Biology, Center for Spatial Analysis, University of Oklahoma, Norman, OK, United States; ^4^College of Urban and Environmental Sciences, Northwest University, Xi’an, China

**Keywords:** elevational gradients, rhizosphere soil, microbial community, plant properties, soil properties

## Abstract

Elevational gradients strongly affect microbial biodiversity in bulk soil through altering plant and soil properties, but the effects on rhizosphere microbial patterns remain unclear, especially at large spatial scales. We therefore designed an elevational gradient experiment to examine rhizosphere microbial (bacteria, fungi and arbuscular mycorrhizal fungi) diversity and composition using Illumina sequencing of the 16S rRNA and ITS genes for comparison to plant and soil properties. Our results showed that bacterial and fungal alpha diversity was significantly higher at mid-elevation, while AMF alpha diversity decreased monotonically. The beta diversities of the three groups were significantly affected by elevational gradients, but the effect on bacterial beta diversity was larger than on fungal and AMF beta diversity. *Proteobacteria*, the dominant phyla of bacteria, was significantly higher at the mid-elevation, while *Acidobacteria* and *Actinobacteria* significantly decreased as elevation increased. The main fungal taxa, *Basidiomycota*, significantly decreased with elevation, and *Ascomycota* significantly increased with elevation. *Glomeromycota*, the dominant AMF phyla, responded insignificantly to the elevational gradients. The responses of bacterial and fungal alpha diversity were mostly associated with tree diversity and organic carbon, whereas AMF alpha diversity mainly depended on litter N and P. Changes in bacterial community composition along the elevational gradient were explained primarily by litter N and P, and litter P was the main driver of fungal and AMF community composition. Overall, our results suggest that plant litter, particularly litter N and P, were the main source of external carbon input and drove the observed differences in rhizosphere microbial diversity and community composition. Our results highlight the importance of litter nutrition in structuring rhizosphere microbial communities in mountain ecosystems.

## Introduction

In mountain ecosystems, many climate factors can vary drastically over a short spatial distance (see review by Körner, 2007), which not only influence aboveground macroorganisms but also microorganisms belowground (Fierer et al., 2011; Sundqvist et al., 2013). Therefore, elevational gradients can be used in experimental design to investigate the ecological responses of the microbial community to changing environments (Bragazza et al., 2015; Siles and Margesin, 2017). To date, a variety of studies have investigated the response of microbial community in bulk soil to elevational gradients, suggesting that elevational gradients strongly affect the microbial diversity and community composition of bulk soils by altering plant and soil characteristics (Meng et al., 2013; Li et al., 2018; Saitta et al., 2018). In such dynamic environments, the shift of the microbial community in bulk soil and its interaction with plants, particularly plant roots, can potentially alter rhizosphere microorganisms. However, few studies have examined the elevational patterns of rhizosphere microorganisms and its drivers, especially in large elevational scales. The lack of research in this area greatly hinders our predictions of nutrient cycling of terrestrial ecosystem under climate warming, despite the importance of rhizosphere microorganisms in mediating biogeochemical cycles (Van der Heijden et al., 2008; Delgado-Baquerizo et al., 2016).

Compared with microbes in bulk soil, rhizosphere microbes are strongly influenced by aboveground plants (Berg and Smalla, 2009; Pii et al., 2016). Plants are broadly perceived to influence rhizosphere microbes through the provision of carbon compounds in root exudates, plant litter, or plant secondary metabolites (Bardgett and Wardle, 2010; Schlatter et al., 2015). Plants can also significantly impact rhizosphere microbes through plant-induced changes to soil properties in the rhizosphere (e.g., N, P) (Herold et al., 2014). In montane forests, the changes in plant and soil properties induced by elevational gradients would inevitably lead to dramatic changes in the rhizosphere microbial community. In particular, shifts in plant communities could result in different microbial assemblages in the rhizosphere due to changes in aboveground (litter) and belowground (root exudation) resource inputs (Kaiser et al., 2015; Zhalnina et al., 2018). For example, arbuscular mycorrhizal fungi (AMF), which play key roles in multitrophic interactions between plants and soils, is strongly influenced by root exudates (Lugo et al., 2008). The dramatic changes in climate and vegetation that occur along the elevational gradient also influence the return rate and quality of litter nutrients (Aponte et al., 2012; Saura-Mas et al., 2012) and indirectly affect the assembly of rhizosphere microbes (Aponte et al., 2011). For example, Lang et al. (2016) found that soil phosphorus in the forest mainly comes from litter decomposition, which shape both plant and microbial communities. However, how elevational gradients affect rhizosphere microbial communities by altering above and below-ground properties is still unclear.

To comprehensively understand the changes in rhizosphere microbial communities along elevational gradient and reveal their biotic and abiotic determinants, we investigated the diversity and composition of different microbial communities (bacteria, fungi, and AMF) in rhizosphere soil along elevational gradients of approximate1300 mon Taibai Mountain. Several studies performed in this elevation range have found patterns in above-plant diversity and microbes in bulk soil (Tang et al., 2012; Ren et al., 2018a). However, the responses of rhizosphere microbial communities to the elevational gradient were not the focus of these studies. Here, we hypothesized that the elevational gradient effects on rhizosphere microbial communities depend primarily on plant and soil properties, and that different microbial communities (bacteria, fungi, and AMF) would respond differently to plant and soil properties. Specifically, we aimed to: (1) reveal how rhizosphere microbial communities respond to elevational gradient; (2) compare how rhizosphere microbial community (bacterial, fungal, and AMF) diversity and composition respond to elevational gradient; and (3) evaluate the effect of plant and soil properties on the rhizosphere microbial community.

## Materials and Methods

### Site Description

**FIGURE 1:**
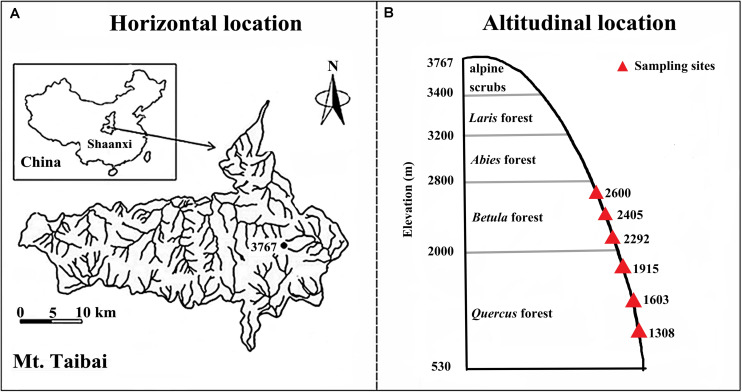
Map of the study area **(A)** and the sampling sites along the elevational gradient **(B)**.

Our study was conducted on Taibai Mountain, Shaanxi Province, China (33°49–34°10′N; 107°19′–107°58′E, Figure 1A), which spans from 530 to 3767 m in elevation and is the highest mountain in the Qinling Mountain range. The Qinling Mountains run east–west in central China and form a transitional zone between northern subtropical and warm-temperate zones, which make it a global biodiversity hotspot (Dang et al., 2007; Guo et al., 2019). Taibai Mountain is in the northern slope of range and is in a warm temperate region. Mean annual temperature and precipitation are 11.4°C and 910.6 mm, respectively. The large span in elevation means that Taibai Mountain hosts the most complete spectrum of vegetation types in the Qingling Mountains. The natural vegetation types along the elevational gradient are oak forests (*Quercus* sp.) (<2200 m), birchforests (*Betula* sp.) (2300–2800 m), fir forests (*Abies* sp.) (2800–3200 m), larchforests (*Larix* sp.) (3000–3400 m), and alpine shrubs (>3400 m) (Ren et al., 2006).

Our experiment was conducted between elevations of 1200–2700 m where the topography was relatively consistent but the vegetation varied with elevation and included *Quercus acutidentata* forest (1200–1800 m), *Quercus liaotungensi* (1900–2300 m), *Betula albosinensis* (2300–2600 m), and *Betula utilis* (2600–2700 m). Within the elevational range, we selected six elevations (1308, 1603, 1915, 2292, 2405, and 2600 m) as our experimental sites (Figure 1B). For each elevation, samples were collected from the three independent replicate plots (50 × 50 m each), the plots were separated by ≥ 13.5 m to obtain independent samples for statistical analysis (Marriott et al., 1997). For each variable, we averaged measurements from the three replicates at the same elevation to represent the observations of the study site. In total, 18 observations were obtained (six elevations × three replicates) for each variable. The experiments were carried out in July 2018.

### Plant Survey and Soil Sampling

For plant investigation, three 10 × 10 m quadrants, five 5 × 5 m quadrants, and ten 1 × 1 m quadrants were randomly selected in each 50 × 50 m plot to determine the composition and richness for tree, shrub, and herb, respectively. For woody plants, the percentage of total base diameter for each family was calculated to reflect the relative importance. For herbaceous plants, the relative cover of each family was calculated to represent the relative importance. The leaves, litter, and fine roots (0–10 cm depth) were collected from major tree species at each elevation to determine their carbon, nitrogen, and phosphorus content. The fine roots were separated from the soil via water bath extraction and with the help of a fine meshed sieve.

Soil was sampled only for the rhizosphere soil, which we defined as soil tightly adhering to the root surface of the dominant tree species. Rhizosphere soil was also collected from the three independent replicate plots for each elevation. For each 50 × 50 m plot, the rhizosphere soil of ten individuals of the dominant tree species were show collected with a sterile soft brush and then were homogenized to represent the rhizosphere soil sample of the plot. After sampling, each soil sample was divided into two subsamples. One subsample was immediately place in an insulated container with ice, transported to the laboratory, and then stored at −80°C for DNA extraction. The other subsample was air-dried after passing through a 2 mm sieve to analyze soil properties.

### Plant and Soil Properties Analysis

We first dried plant samples (leaves, roots, and litter)to a constant weight in an oven at 60°C and then determined their C, N, and P contents after grinding finely to 0.15 mm with a ball mill (Wang et al., 2015). The C content was determined using the K_2_Cr_2_O_7_ oxidation method, and the N and P contents of the digested solution were determined using the Kjeldahl and colorimetric (UV spectrophotometer) methods, respectively (Lin et al., 2011). For rhizosphere soil samples, the Walkley-Black method (Nelson et al., 1982), the Kjeldahl method (Bremner and Mulvaney, 1982), and the colorimetric method after wet digestion with H_2_SO_4_ + HClO_4_ were used to determine soil organic carbon (SOC), total nitrogen (TN), and total phosphorus (TP), respectively.

### DNA Extraction and Sequencing

Following the manufacturer’s instructions, soil (0.5 g fresh weight) was extracted using the FastDNA Spin Kit (MP Biomedicals, Cleveland, United States). The concentration and quality of the DNA were evaluated by a NanoDrop 2000 spectrophotometer (Thermo Fisher Scientific, Wilmington, DE, United States). PCR of the bacterial 16S rRNA gene targeting the V4 region was amplified using primers 515F (5′-GTGCCAGCMGCCGCGG-3′) and 907R (5′-CCGTC AATT CMTTTRAGTTT-3′) (Biddle et al., 2008). The amplification of the fungal ITS-1 region was achieved using primers ITS1F (50-ACTTGGTCATTTAGAG-GAAGTAA-30) and ITS2 (50-BGCTGCGTTCTTCATCGATGC-30) (Mukherjee et al., 2014). Partial small subunit (SSU) ribosomal RNA gene fragments of arbuscular mycorrhizal fungi were amplified using nested PCR (Borriello et al., 2012), with the universal eukaryotic primers NS1 and NS4(White et al., 1990), and a subsequent amplification round with the Glomeromycota-specific primers AML1 and AML2 (Jaikoo et al., 2010). The PCR products were extracted from 2% agarose gels and purified using the AxyPrep DNA Gel Extraction Kit (Axygen Bio-sciences, Union City, CA, United States) according to the manufacturer’s instructions and quantified using QuantiFluor^TM^ −ST (Promega, United States.). Finally, an equal amount of PCR product from each sample was sent for pyrosequencing using the Illumina’s MiSeq platform at Personal Biotechnology Co., Ltd. Shanghai, China.

For all sequencing reads, raw fastq files were demultiplexed, quality-filtered using following criteria: (i) the reads were truncated with an average quality score < 20 in a 50 bp sliding window; (ii) primers were exactly matched allowing two nucleotide mismatches, and reads containing ambiguous bases were removed; (iii) sequences with > 10 bp overlap were merged according to their overlap sequence. Quantitative Insights Into Microbial Ecology pipeline software (QIIME) software (v1.8.0)^1^ was used to obtain 16S rRNA operational taxonomic units (OTUs) (Caporaso et al., 2012). Sequence analysis was performed using the USEARCH v5.2.32 to filter and eliminate noise from the data by clustering similar sequences with more than 97% similarity. Finally, the complete dataset was sent to the Sequence Read Archive (SRA) database of the National Center for Biotechnology Information (NCBI) under Accession NO. SRP223550 for bacteria, SRP223554 for fungi and SRP223556 for AMF.

### Statistical Analyses

We used species richness, Shannon index, and Simpson’s index (1/D) to estimate the alpha diversity of the plant community and the Shannon index to calculate the alpha diversity of rhizosphere microbial communities (bacterial, fungal and AMF). In order to investigate the relationships between biological communities and elevational gradient, a non-metric multidimensional scaling (NMDS) analysis was conducted for the plant and rhizosphere microbial communities based on Bray-Curtis distances. In addition, we used the analysis of similarities (ANOSIM) to determine the significance of separation along the climate gradients (Clarke et al., 2014). The alpha diversity index was calculated using Mothur software (Schloss et al., 2009). NMDS and ANOSIM were performed using the“vegan” package of R program (R Core Team, 2016).

To further investigate the possible pathways through which plant communities influence the composition of the rhizosphere microbial community directly and indirectly along the spatial gradients, we preformed partial least squares path modeling (PLS-PM). Through PLS-PM analysis, the observed variables can be explained by the latent variables (Sanchez, 2013). In our study, the latent variables included elevation, plant properties, soil properties, and rhizosphere microbial communities (bacterial, fungal and AMF). Each latent variable could include at least one observed variable. In the PLS-PM, the direction and strength of linear relationships between latent variables was represented by the path coefficients and the explained variability (R^2^) was also estimated. The effective model was evaluated by the average variance extracted (AVE) and the composite reliability (CR). When the AVE and CR are higher than 0.5 and 0.7, respectively, this model is acceptable. This analysis was conducted using PLS, provided by the Smart PLS 2.0 M3 software. In our study, both the AVE and CR fit these standards (AVE > 0.5; CR > 0.7) (Vanalle et al., 2017).

Furthermore, the relationships between the rhizosphere microbial characteristics and the plant and soil properties were determined using spearman correlation analysis. Correlations between the rhizosphere microbial compositions and the plant and soil properties were determined using redundancy analysis (RDA) (Clarke et al., 2014). The one-way analysis of variation (ANOVA) was used to test the effect of elevation gradients on plant and soil characteristics, rhizosphere microbial diversity, and dominant phyla. The analysis were conducted using the “vegan” package of R program. *P* < 0.05 was considered statistically significant.

## Results

### Plant and Soil Properties Along the Elevational Gradient

**FIGURE 2:**
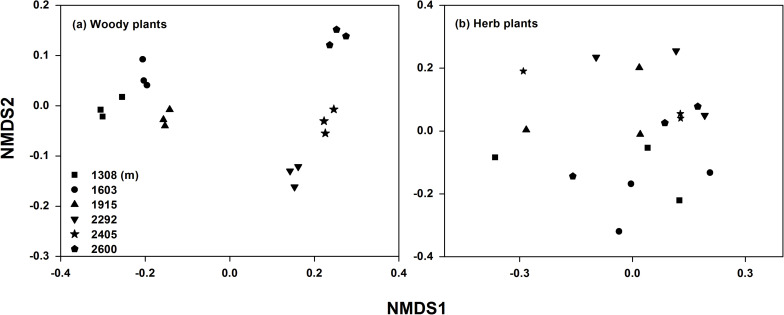
Non-metric multidimensional scaling (NMDS) analysis of woody **(a)** and herb **(b)** plant community compositions along the elevational gradient.

Plant and soil characteristics were affected greatly by elevational gradient but showed different trends (Table 1). For plant communities, the alpha diversity (richness, Simpson, and Shannon index) of trees and shrubs were significantly higher at mid-elevation, while the alpha diversity of herb community had no significant trend with elevation. The species composition of the plant communities also changed along the elevational gradient (Supplementary Table S1). Particularly for woody plant community, the NMDS ordination showed a clear separation of sites based on the species composition along the elevation (Figure 2). In addition, leaf C, leaf N, leaf P, litter C, root C, and root N were significantly higher at mid-elevation. Litter N increased with elevation, and litter P was higher at low elevation. For rhizosphere soil characteristics, SOC and TN were also higher at mid-elevation. However, root P and soil TP did not significantly change with the elevation.

**TABLE 1:** Plant and soil properties along the elevational gradient.

Properties	Elevational gradient (m)						F	*P*
	1308	1603	1915	2292	2405	2600		
Trichness	6.67 ± 0.58B	8 ± 1AB	10 ± 1A	8 ± 1AB	6 ± 1B	6 ± 1B	8.05	**0.002**
Tsimpson	2.54 ± 0.05B	3.14 ± 0.30B	4.78 ± 0.78A	2.94 ± 0.29B	2.84 ± 0.18B	2.94 ± 0.28B	12.71	**<0.001**
Tshannon	1.13 ± 0.12C	1.37 ± 0.07B	1.69 ± 0.1A	1.32 ± 0.05BC	1.2 ± 0.02BC	1.26 ± 0.07BC	18.70	**<0.001**
Srichness	7 ± 1D	8 ± 1D	12 ± 0.2C	18 ± 1A	15 ± 1B	8 ± 1D	59.6	**<0.001**
Ssimpson	4.88 ± 0.32B	4.13 ± 0.36B	6.35 ± 1.90AB	4.82 ± 0.84B	8.14 ± 0.69A	6.09 ± 0.36AB	8.19	**0.001**
Sshannon	1.55 ± 0.08C	1.49 ± 0.05C	1.98 ± 0.4AB	2.13 ± 0.12A	2.08 ± 0.13AB	1.86 ± 0.11B	24.12	**<0.001**
Hrichness	6 ± 1D	16 ± 1A	9 ± 1C	12 ± 1B	13 ± 1B	11 ± 1BC	35.30	**<0.001**
Hsimpson	6.96 ± 0.53A	6.29 ± 0.22AB	4.21 ± 0.25C	5.16 ± 0.16BC	4.95 ± 0.09BC	5.77 ± 0.56B	23.58	**<0.001**
Hshannon	1.7 ± 0.03C	2.05 ± 0.*A*	1.69 ± 0.7C	1.93 ± 0.05AB	1.88 ± 0.02B	1.84 ± 0.06BC	21.45	**<0.001**
Leaf C	461.12 ± 14.03B	490.38 ± 14.87AB	515.87 ± 24.66A	494.14 ± 12.35AB	481.21 ± 7.36AB	474.44 ± 16.56AB	4.57	**0.015**
Leaf N	31.66 ± 2.82BC	31.59 ± 1.07BC	39.75 ± 1.55A	35.24 ± 1.39B	26.78 ± 0.41C	30.7 ± 1.5C	22.14	**<0.001**
Leaf P	2.27 ± 0.14AB	2.26 ± 0.18AB	2.76 ± 0.1A	2.8 ± 0.34A	1.93 ± 0.34B	2.27 ± 0.3AB	10.21	**<0.001**
Litter C	408.96 ± 7.45B	425.85 ± 10.39B	484.63 ± 4.*A*	486.09 ± 21.15A	477.56 ± 0.2A	424.29 ± 8.63B	31.61	**<0.001**
Litter N	17.76 ± 0.54C	19.18 ± 0.57C	21.83 ± 0.57B	20.93 ± 1.25BC	24.55 ± 0.33A	22.83 ± 0.43AB	39.22	**<0.001**
Litter P	6.48 ± 0.21B	7.5 ± 0.46A	3.14 ± 0.12C	2.06 ± 0.11D	2.16 ± 0.27D	1.59 ± 0.44D	213.10	**<0.001**
Root C	454.83 ± 4.14B	481.73 ± 6.62B	504.89 ± 11.17AB	516.83 ± 20.5A	478.62 ± 9.71B	453.44 ± 8.61B	15.35	**<0.001**
Root N	9.43 ± 0.11B	10.29 ± 0.53B	12.65 ± 0.5A	12.54 ± 1.21A	10.26 ± 0.11B	8.94 ± 0.57B	18.87	**<0.001**
Root P	1.6 ± 0.14A	1.56 ± 0.13A	1.54 ± 0.14A	1.46 ± 0.31A	1.62 ± 0.14A	1.66 ± 0.1A	0.52	0.756
SOC	56.03 ± 0.59C	69.41 ± 1.07B	85.71 ± 3.63A	81.19 ± 1.2A	59.31 ± 1.07C	49.86 ± 0.14D	214.90	**<0.001**
TN	4.57 ± 0.05F	5.23 ± 0.05D	6.96 ± 0.11A	6.5 ± 0.03B	6.46 ± 0.19B	5.78 ± 0.21C	143.70	**<0.001**
TP	0.34 ± 0.03A	0.4 ± 0.05A	0.44 ± 0.03A	0.47 ± 0.09A	0.46 ± 0.11A	0.46 ± 0.06A	1.90	0.168

*Different letters indicate significant differences (ANOVA, P < 0.05, Tukey’s HSD post hoc analysis) among different elevation gradients. Trichness, tree richness; Tsimpson, tree simpson index; Tshannon, tree shannon index; Srichness, shrub richness; Ssimpson, shrub simpson index; Sshannon, shrub Shannon index; Hrichness, herb richness; Hsimpson, herb simpson index; Hshannon, herb shannon index; SOC, soil organic carbon; TN, total nitrogen; TP, total phosphorus.*

### Microbial Diversity and Composition Along the Elevational Gradient

After quality sequencing, a total of 1,154,395 bacterial sequences, 1,321,332 fungal sequences and 1,614,413 AMF sequences were identified from the 18 soil samples. For bacteria, there were 48,477–76,746 sequences per sample, with a mean of 64,133 sequences. For fungi, the number of sequences ranged from 49,297 to 129,882 per sample, with a mean of 73,407 sequences. For AMF, the sequences varied from 80,476 to 105,892 per sample, with a mean of 89,689 sequences. To minimize any bias in the distribution of taxa, bacterial, fungal and AMF diversity of each treatment were calculated based on randomly selected sequence until the count reached saturation in the rarefaction curves. For the downstream analysis of bacteria, datasets were rarefied to 48,000 sequences. For downstream analysis of fungi, datasets were rarefied to 49,000 sequences. For downstream analysis of AMF, datasets were rarefied to 70,000 sequences.

**FIGURE 3:**
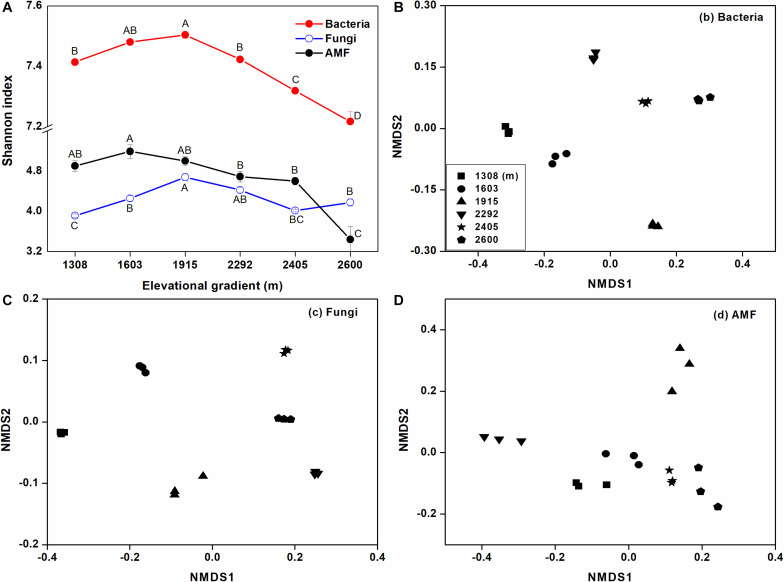
The effects of elevational gradient on microbial (bacterial, fungal, and AMF) alpha **(A)** and beta diversity **(B–D)** in rhizosphere soil. The bar represents standard deviation (SD). The beta diversity is represented by unweighted UniFrac distances based on the relative abundances of OTUs.

Based on the Shannon index, rhizosphere microbial alpha diversity significantly changed with elevational gradient, and elevational gradient had more of an impact on bacterial alpha diversity than fungal and AMF alpha diversity (Figure 3A). More specifically, bacterial and fungal alpha diversity were significantly higher at mid-elevation, while AMF alpha diversity declined as elevation increased. Furthermore, the NMDS ordination reflected a clear separation of sites along the elevation gradient for the rhizosphere bacterial and AMF communities (Figures 3B,D). For fungi, the degree of aggregation of the sample sites at the lower elevation was distant but not for sites at higher elevation (Figure 3C). Analysis of similarities (ANOSIM) also showed that the influence of the elevational gradient on bacterial beta diversity (*R* = 0.90, *p* < 0.01) was higher than its influence on fungal beta diversity (*R* = 0.69, *p* < 0.01) and AMF beta diversity (*R* = 0.61, *p* < 0.01).

**FIGURE 4:**
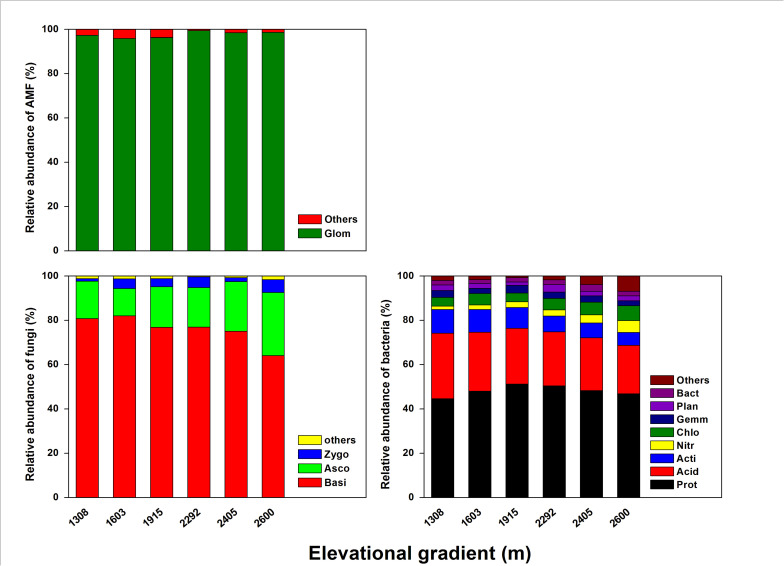
The effects of elevation gradient on the bacterial, fungal, and AMF taxonomic compositions in rhizosphere soil. These are the abbreviations of microbial taxa: *Proteobacteria* (Prot), *Acidobacteria* (Acid), *Actinobacteria* (Acti), *Nitrospirae* (Nitr), *Chloroflexi* (Chlo), *Gemmatimonadetes* (Gemm), *Planctomycetes* (Plan), *Bacteroidetes* (Bact), *Basidiomycota* (Basi), *Ascomycota* (Asco), *Zygomycota* (Zygo), *Glomeromycota* (Glom).

As for rhizosphere microbial composition (Figure 4), most of the bacterial community was *Proteobacteria* (44.58–52.25%), *Acidobacteria* (21.89–29.61%), and *Actinobacteria* (5.8–10.68%), with *Chloroflexi*, *Gemmatimonadetes*, *Planctomycetes*, and *Bacteroidetes* each accounting for ≤ 5.0% (Supplementary Table S2). Of those bacteria, Proteobacteria was significantly more abundant at the mid-elevation. *Acidobacteria* and *Actinobacteria* both significantly decreased with elevation, whereas *Nitrospirae* and *Chloroflexi* increased with elevation. For the fungal community, the dominant phyla were *Basidiomycota* (64.08–82.05%), *Ascomycota* (12.36–28.53%), *Zygomycota* (1.14–5.81%), and a small proportion of unidentified fungi (0.33–1.58%) (Supplementary Table S3). *Basidiomycota* significantly decreased with elevation and *Ascomycota* significantly increased with elevation. The AMF community was dominated by *Glomeromycota* (96.29–99.25%) and a small proportion of the community was unidentified (≤ 5.0%) (Supplementary Table S4). However, the composition of the AMF community did not significantly change with the elevation.

### Effect of Plant and Soil Properties on Microbial Diversity and Composition

**FIGURE 5:**
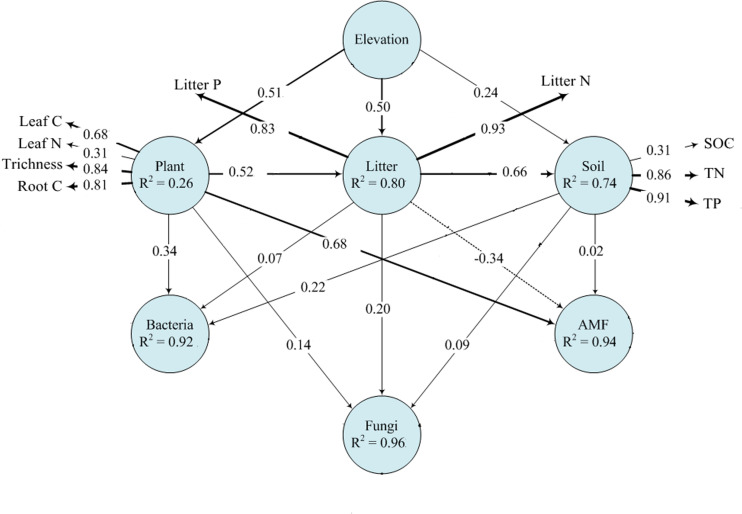
Effects of elevation on microbial communities by plant and soil properties based on PLS-PM analysis. The width of arrows is proportional to the strength of path coefficients. Continuous and dashed arrows indicate positive and negative relationships, respectively. R^2^ denotes the proportion of variance explained.

Plant and soil properties had significant relationships with rhizosphere microbial diversity (Table 2). Among the plant properties, tree alpha diversity was the most significant attribute affecting bacterial and fungal alpha diversity, while AMF alpha diversity was mostly affected by litter N and litter P. Litter N and litter P were the most significant attributes affecting bacterial and fungal beta diversity, while AMF beta diversity was mostly affected by leaf N and leaf P. Among the soil properties, SOC was the most significant attribute affecting bacterial, fungal, and AMF alpha diversity. Soil TN and TP significantly affected bacterial and fungal beta diversity, and AMF beta diversity was not affected by soil properties. Our PLS-PM analysis showed that rhizosphere bacteria were affected by plant and soil properties together, while fungi and AMF was affected mainly by plant and litter properties and weakly influenced by soil properties (Figure 5).

**TABLE 2:** The relationships of microbial diversity with plant and soil properties based on spearman rank correlation analysis.

Properties	Bacterial diversity			Fungal diversity			AMF diversity		
	Shannon	NMDS1	NMDS2	Shannon	NMDS1	NMDS2	Shannon	NMDS1	NMDS2
Trichness	**0.79^c^**	–0.08	0.27	**0.73^c^**	–0.36	–0.47	**0.59^a^**	0.42	0.12
Tsimpson	0.46	**0.50^a^**	–0.09	**0.73^c^**	0.14	−**0.80^c^**	0.26	0.26	–0.45
Tshannon	**0.65^b^**	0.25	0.19	**0.83^c^**	–0.12	−**0.56^a^**	0.46	0.34	–0.21
Srichness	0.04	–0.01	−**0.51^a^**	0.11	0.35	–0.28	0.09	–0.41	–0.04
Ssimpson	–0.20	−**0.57^a^**	0.22	−**0.60^b^**	–0.52	**0.73^a^**	0.10	–0.23	**0.55^b^**
Sshannon	0.01	–0.18	−**0.51^a^**	0.08	0.22	–0.05	0.07	–0.23	0.15
Hrichness	0.05	0.23	−**0.59^b^**	0.46	**0.55^a^**	–0.45	–0.24	0.31	–0.25
Hsimpson	–0.41	**0.61^b^**	–0.24	–0.06	**0.72^c^**	–0.17	−**0.56^a^**	–0.14	−**0.65^b^**
Hshannon	–0.22	0.40	–0.54	0.33	**0.70^c^**	–0.29	–0.47	0.39	–0.39
Leaf C	**0.56^a^**	0.28	–0.12	**0.75^c^**	0.05	−**0.77^c^**	0.33	0.32	–0.23
Leaf N	**0.65^b^**	0.06	0.16	**0.71^c^**	–0.41	–0.39	0.31	**0.78^c^**	0.01
Leaf P	**0.47^a^**	0.01	0.07	**0.65^b^**	–0.22	–0.31	0.18	**0.70^c^**	0.04
Litter C	0.28	0.38	–0.28	**0.67^b^**	0.41	−**0.60^b^**	–0.01	0.38	–0.39
Litter N	−**0.50^a^**	**0.78^c^**	−**0.53^a^**	0.12	**0.95^c^**	–0.29	−**0.62^b^**	–0.20	−**0.81^c^**
Litter P	**0.65^b^**	−**0.66^b^**	**0.63^b^**	–0.12	−**0.79^c^**	–0.05	**0.85^c^**	–0.28	**0.68^b^**
Root C	**0.57^a^**	0.09	–0.11	**0.76^c^**	0.10	−**0.61^b^**	0.33	0.45	–0.09
Root N	**0.59^a^**	0.03	–0.10	**0.72^c^**	0.08	−**0.70^c^**	0.32	0.43	–0.03
Root P	–0.29	0.10	0.03	–0.27	0.18	0.25	–0.12	–0.24	–0.20
SOC	**0.79^c^**	–0.04	0.09	**0.76^c^**	–0.13	−**0.70^c^**	**0.54^a^**	0.43	0.08
TN	0.25	**0.62^b^**	–0.24	**0.72^c^**	**0.51^a^**	−**0.67^b^**	–0.10	0.39	−**0.61^b^**
TP	–0.07	**0.61^b^**	–0.45	0.45	**0.59^a^**	–0.34	–0.36	0.10	−**0.63^b^**

*^a,b,c^ represent that the correlation is significant at P = 0.05, 0.01, and 0.001, respectively.*

**FIGURE 6:**
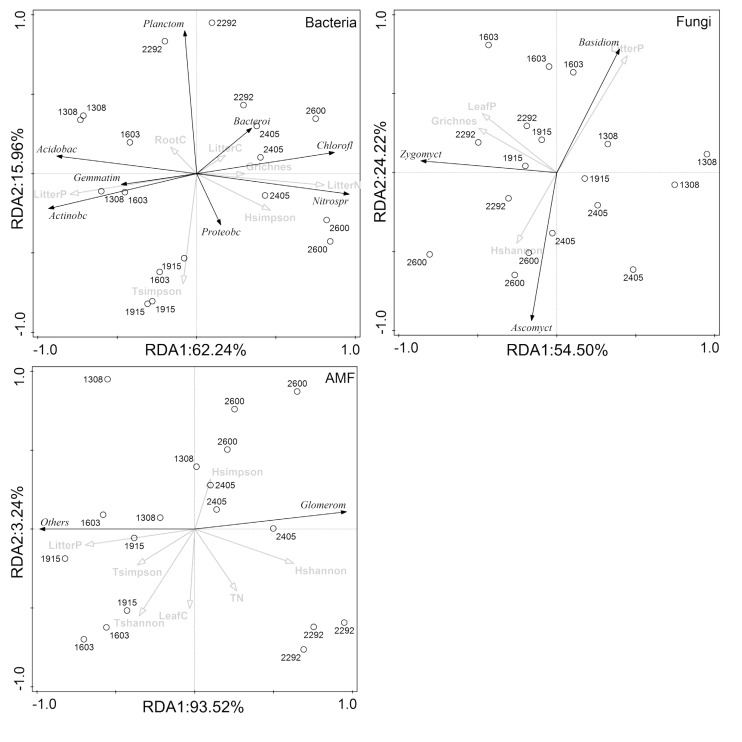
Redundancy analysis (RDA) ordination to identify the relationships between microbial phyla and plant and soil properties.

RDA demonstrated that the composition of the rhizosphere microbial community at the phylum level was largely affected by plant properties rather than soil properties (Figure 6). Plant properties, particularly litter P, were responsible for much of the variations in the composition of all rhizosphere microbial taxa (bacteria, fungi, and AMF). Litter N, litter P, and tree Simpson index were significantly related to changes of *Chloroflexi*, *Nitrospirae*, *Acidobacteria*, *Actinobacteria*, *Gemmatimonadetes*, and *Planctomycetes* in the bacterial community. Litter P and shrub richness were associated with changes of *Basidiomycota*, *Ascomycota*, and *Zygomycota* in the fungal community, and litter P was correlated with *Glomeromycota* and other phylum in the AMF community.

## Discussion

### Differences in Rhizosphere Bacterial, Fungal, and AMF Diversity Along the Elevation Gradient

Elevational gradients lead to shifts in plant and soil properties, and then cause the microbial diversity in rhizosphere soils to change (Berg and Smalla, 2009). However, the effects of plant and soil properties on diversity patterns of rhizosphere microbes along the elevation remain unclear. By studying the rhizosphere microbial community and corresponding plant and soil properties along a 1300 m elevational range on Taibai Mountain, a global biodiversity hot spot, we found that rhizosphere microbial diversity changed significantly along the elevational gradient depending on plant and soil properties.

In this study, both rhizosphere bacterial and fungal alpha diversity were higher at mid-elevation. The diversity of bacterial community observed was consistent with the pattern reported by Ren et al. (2018a), which investigated bulk soil microorganisms on Taibai Mountain. However, Ren et al. (2018a) found that the elevational gradient had no effects on fungal alpha diversity in bulk soil. This suggested that rhizosphere fungal diversity, compared with rhizosphere bacterial diversity, was more influenced by aboveground vegetation. In general, rhizosphere soil has a higher nutrient and moisture content than bulk soil due to root activities (Kielak et al., 2008; Ai et al., 2012). Guo et al. (2016) found that the diversity of the rhizosphere fungal community was generally higher than in bulk soil. In addition, most fungi are dependent on a particular host plant species or genera (Grayston et al., 1998). A great number of studies have shown a positive relationship between fungal richness and plant richness (Hiiesalu et al., 2014; Tedersoo et al., 2014, 2016). For example, Saitta et al. (2018) found that fungal richness was strongly associated with tree richness along an elevational gradient. In agreement with these studies, our study also found that tree alpha diversity was the most significant attribute affecting fungal alpha diversity among the plant and soil properties (Table 2).

In contrast to the bacterial and fungal communities, the AMF community exhibited a monotonically decreasing pattern along the elevational gradient. This result was consistent with other published studies, such as Lugo et al. (2008) and Gai et al. (2012), which showed similar trends in AMF diversity along elevational gradients. However, these studies did not investigate the drivers of these changes in the AMF assemblages. In our study, the change in AMF diversity along the elevational gradient was highly related to litter P (Table 2). In forest ecosystems, most phosphorus is sequestrated in plant litter, which limits its availability for plants. In this situation, AMF can improve phosphorus availability for plants and simultaneously obtain carbon from root exudates (Landis and Fraser, 2008; Lugo et al., 2008). Therefore, litter P plays a more important role in regulating AMF diversity than other properties.

Moreover, elevational gradients also showed significant effects on rhizosphere microbial beta diversity (Figure 2). For the bacterial and fungal community, their beta diversity was most closely correlated with litter N and P (Table 2). This correlation probably arises because changes in nutrient availability, due to differing return rates and litter nutrient quality along the climate and vegetation gradient, ultimately lead to differences in the rhizosphere bacterial and fungal community (Jacob et al., 2009). In addition, rhizosphere soil TN and TP were also responsible for differences in bacterial and fungal beta diversity, which further indicated that the availability of N and P drive the changes in the rhizosphere bacterial and fungal communities along the elevational gradient. In contrast, AMF beta diversity responded significantly to leaf N and P. This response is probably due to the selective effects of host plant root exudates on a specific AMF population (Hartmann et al., 2009). Leaf N and P reflect the nutrient uptake and metabolic strategies of plants (Richardson et al., 2009). Previous studies have suggested that AMF was correlated with plant metabolic type (Hetrick et al., 1990; Lugo and Cabello, 2002). Therefore, leaf N and leaf P can explain the change in AMF beta diversity.

In addition, analysis of the community alpha and beta diversity response to elevational gradient revealed that bacteria varied more than fungi and AMF (Figure 2). This result agreed with other previous reports that bacterial communities is more sensitive to changes in climate, such as rainfall and temperature (Yuste et al., 2011), than fungal communities (Ren et al., 2018b). The different responses were associated with plant and soil properties. Compared with rhizosphere bacteria, rhizosphere fungi and AMF were more affected by plant properties, which may offset the direct effect of elevational gradients (Susan et al., 2015). Overall, these results satisfied our hypothesis that different rhizosphere microbial taxa respond differently to elevational gradients. Plant properties had more significant effects on rhizosphere microbial diversity than soil properties, particularly for the AMF community.

### Differences in Rhizosphere Bacterial, Fungal, and AMF Community Compositions Along the Elevation Gradient

In addition to the effect of plant and soil characteristics on rhizosphere microbial diversity, the influence of these properties on the community composition of rhizosphere microbial taxa has also been seldom reported. Here, we found that elevational gradients had different effects on the compositions of bacterial, fungal, and AMF communities. Such differential responses can be largely explained by plant litter N and litter P rather than soil properties (Figure 4), which further indicate that plant properties were the main driver for the changes of the rhizosphere microbial structure.

For the bacterial community, *Proteobacteria* was most abundant at mid-elevation and was largely affected by tree Simpson index (Figures 2, 4), which might be because *Proteobacteria* prefers available carbon (Fierer et al., 2007). Highly diverse tree communities have substantially greater rhizosphere resource input by root exudation, and therefore may promote *Proteobacteria*. In addition, *Proteobacteria* was one of the most diverse phyla in the bacterial community, and higher soil resource concentrations and diversity can modulate competitive species interactions within the phylum and thus promote their abundance (Schlatter et al., 2015). *Acidobacteria* and *Actinobacteria* were most abundant at low-elevation and were mostly related to changes in litter N and litter P (Figures 2, 4). This relationship is probably because higher litter P and lower litter N at lower elevation restrict litter decomposition and nutrient return, and result in lower resource availability (Aponte et al., 2012; Saura-Mas et al., 2012). *Acidobacteria* and *Actinobacteria* have been found to adapt to acidic and resource-limited conditions (Stroobants et al., 2014; Siles and Margesin, 2016). Therefore, higher litter P and lower litter N promote their abundance. Other specific taxa, mainly *Nitrospirae* and *Chloroflexi*, were also most closely related to litter N and litter P (Figure 5), and these patterns were likely due to their ecological strategies (Sorokin et al., 2012; Siles and Margesin, 2016). Therefore, these results suggested that the different responses of rhizosphere bacterial community composition to the elevational gradients were more dependent on plant characteristics than soil characteristics.

The fungal community composition was largely explained by litter P (Figure 5), which is in line with Marschner et al. (2006), who reported that fungal community composition in the rhizosphere was affected by P availability since P availability in the forest largely depended on the return of litter P. In addition, He et al. (2016) suggested that available P was a key parameter that determines the diversity and composition of the fungal community, and other soil parameters played secondary roles. However, the dominant fungal phyla, *Basidiomycota* and *Ascomycota*, responded oppositely to the change in litter P (Figure 4), which might be caused by their different responses to elevational change. *Basidiomycota* decreased with the elevational gradient, *Ascomycota* while increased with the elevational gradient (Supplementary Table S3). The different response between *Basidiomycota* and *Ascomycota* might be attributed to their different ability to decompose litter. As the primary decomposers of dead plant biomass, many studies have found that fungal communities changes during litter decomposition (Osono, 2007; Herzog et al., 2019). In particular, Ascomycota phylum are found to have higher relative abundances during the early stages of decomposition because they have a limited ability to decompose complex organic litter (Osono and Takeda, 2001; Vorísková and Baldrian, 2013),while *Basidiomycota* phylum are relative more abundant during the later stages of decomposition because of their capability to synthesize enzymes required for the degradation of complex polymers (Hannula et al., 2010; Vorísková and Baldrian, 2013). In montane system, litter decomposition would decrease across the elevation gradient (Wang et al., 2010). Therefore, *Basidiomycota* have a higher relative abundance in lower elevation, while *Ascomycota* have a higher relative abundance in higher elevation.

As with the fungal community, the variation in AMF phylum composition can be largely explained by their close relationships with litter P. As the dominant phylum, *Glomeromycota* was negatively related with litter P, which might be due to the function of *Glomeromycota* in the plant-AMF interaction. P uptake is considered the main role of AMF symbiosis (Richardson et al., 2009). In order to acquire more P, under P-limiting conditions the plant will invest more C to increase *Glomeromycota* (Landis and Fraser, 2008). Camenzind et al. (2014) found that elevated P availability reduced AMF abundance significantly. Thus, *Glomeromycota* was more abundant in the low litter P environment. Together with our findings on bacterial and fungal community composition responses, our results showed that the rhizosphere microbial community composition was primarily driven by changes in litter N and P along the elevational gradients. In light of the importance of litter properties for rhizosphere microbial assembly, and litter chemistry rather than the plant species composition *per se*, should receive more attention in future studies that investigate plant-microbe interactions in the rhizosphere.

## Conclusion

We found different responses of rhizosphere microbial diversity and community composition to elevational gradient depending on plant and soil properties. In particular, the changes of rhizosphere microbes along the elevational gradient were more explained by plant properties than soil properties. Rhizosphere bacterial and fungal alpha diversity was higher at mid-elevation sites and was mainly dependent on the dynamics of tree diversity and SOC. Rhizosphere AMF diversity was higher at the low-elevation sites and was mainly dependent on the dynamics of litter N and P. The changes in bacterial community composition were mainly dependent on litter N and P, while the changes in fungal and AMF community composition were mainly dependent on litter P. These results highlight the importance of plant litter nutrition in regulating rhizosphere microbial communities in montane forest, and provide insights into the elevational pattern and drivers of rhizosphere microbial community in montane ecosystems.

## Data Availability Statement

All datasets generated for this study are included in the article/Supplementary Material.

## Author Contributions

YG and FZ conceived and designed the experiments. FZ, YG, CR, and JY performed field work and the experiments. YG analyzed the data and wrote the manuscript. RD and CR revised the manuscript. All authors contributed to the article and approved the submitted version.

## Conflict of Interest

The authors declare that the research was conducted in the absence of any commercial or financial relationships that could be construed as a potential conflict of interest.

## Footnotes

1 http://qiime.org/
