# Supplementary material for: Contrasting Responses of Rhizosphere Bacteria, Fungi and Arbuscular Mycorrhizal Fungi Along an Elevational Gradient in a Temperate Montane Forest of China
Source: Front Microbiol. 2020 Aug 20;11:2042. doi: 10.3389/fmicb.2020.02042 (PMC7469537; doi:10.3389/fmicb.2020.02042)
Supplement: Supplementary file 1 [file Table_1.docx]

**Table S1** Relative covers of plant compositions along the elevation gradient. Different letters indicate significant differences (ANOVA, P < 0.05, Tukey’s HSD post-hoc analysis) among different elevation.

| **Family** | **Elevational gradient (m)** | | | | | | **F** | ***p*** |
| --- | --- | --- | --- | --- | --- | --- | --- | --- |
|  | **1308** | **1603** | **1915** | **2292** | **2405** | **2600** |  |  |
| **Woody plants** |  |  |  |  |  |  |  |  |
| Betulaceae | 2.17±1.84D | 1.98±0.5D | 12.65±1.67C | 24.53±2.12B | 36.52±3.7A | 39.16±5.81A | 79.97 | **<0.001** |
| Pinaceae |  | 1.27±0.95C | 3±1.13BC | 6.65±1.98AB | 6.97±1.44AB | 7.52±2.14A | 9.49 | **0.002** |
| Aceraceae | 10.24±1.24A | 11.78±0.62A | 5.94±1.14B | 3.76±0.86B | 4.77±1.19B | 6.09±1.9B | 17.93 | **<0.001** |
| Salicaceae |  |  | 0.3±0.27B | 5.18±1.26A | 3.48±2.68A | 0.74±0.22B | 7.194 | **0.012** |
| Ericaceae |  |  |  | 1.35±1B | 0.81±0.05B | 23.24±5.39A | 49.03 | **<0.001** |
| Caprifoliaceae | 2.69±1.17C | 8.59±0.43B | 3.82±1.92C | 10.12±2.37B | 19.45±2.44A | 13.38±2.12B | 31.50 | **<0.001** |
| Rosaceae | 9.1±1.74C | 15.31±0.9B | 15.96±1.68B | 26.3±3.31A | 22.61±1.53A | 9.87±2.42C | 31.22 | **<0.001** |
| Tiliaceae |  |  | 1.61±0.68B | 10.41±2.38A | 3.02±1.34B |  | 25.43 | **0.001** |
| Fagaceae | 57.95±5.38A | 47.44±1.42B | 50.13±1.49B | 8.58±0.8C | 2.37±1.35C |  | 271.2 | **<0.001** |
| Cornaceae | 6.25±4.8 | 4.78±2.06 | 4.63±0.82 | 3.12±1.25 |  |  | 0.72 | 0.57 |
| Lauraceae | 3.58±1.15AB | 8.43±4.75A | 1.96±1.91B |  |  |  | 6.41 | **0.03** |
| Anacardiaceae | 5.62±1.15A | 0.15±0.26B |  |  |  |  | 40.28 | **0.003** |
| Ulmaceae | 2.42±2.95 | 0.26±0.22 |  |  |  |  | 1.79 | 0.25 |
| **Herb plants** |  |  |  |  |  |  |  |  |
| Pyrolaceae | 13.33±11.55 | 1.31±2.26 |  |  | 0.61±1.05 | 3.4±5.88 | 2.51 | 0.15 |
| Liliaceae | 28±30.2 | 7.05±5.33 | 10.11±2.33 |  | 5.45±9.45 | 4.6±4.01 | 1.34 | 0.32 |
| Ranunculaceae | 8.88±9.66 | 2.53±4.38 | 9.64±13.45 | 8.68±6.65 | 4.7±4.55 | 9.33±14.08 | 0.28 | 0.91 |
| Labiatae | 1.33±2.31 | 3.52±3.98 | 1.11±1.92 | 10.24±8.17 | 36.14±55.46 | 5.63±5.08 | 1.01 | 0.45 |
| Cyperaceae | 22.98±20.66 | 13.34±12.33 | 11.67±20.21 | 23.31±40.37 | 26.7±22.36 | 21.98±19.32 | 0.18 | 0.96 |
| Asteraceae | 1.33±2.31 | 16.1±23.64 | 7.19±12.45 | 4.55±6.43 | 7.29±8.66 | 0±0 | 0.89 | 0.51 |
| Rosaceae | 1.33±2.31 | 18.39±16.44 | 0.56±0.96 | 2.18±3.78 | 2.87±2.58 | 5.67±9.83 | 2.06 | 0.14 |
| Onagraceae |  | 1.8±3.12 |  |  |  |  |  |  |
| Geraniaceae |  | 0.9±1.56 |  |  |  |  |  |  |
| Gramineae | 16.67±28.87 | 26.64±17.59 |  |  |  | 11.8±12.87 | 0.39 | 0.69 |
| Violaceae |  | 1.32±1.35 |  |  |  |  |  |  |
| Berberidaceae | 1.75±3.04 |  | 30.97±28.55 |  | 1.52±2.62 |  | 3.11 | 0.12 |
| Saxifragaceae |  | 1.08±0.99 | 3.53±3.35 | 5.21±9.02 | 25.4±27.72 | 7.18±4.87 | 1.60 | 0.24 |
| Umbelliferae |  | 0.42±0.73 | 10.19±6.46 | 21.71±13.25 | 13.6±14.44 | 21.1±15.03 | 1.77 | 0.21 |
| Brassicaceae |  |  |  | 10.76±9.34 |  |  |  |  |
| Caryophyllaceae |  | 2.61±4.53 | 5.23±9.06 | 3.77±6.53 |  |  | 0.11 | 0.91 |
| Urticaceae |  | 1.31±2.26 |  | 0.6±1.03 |  |  | 0.25 | 0.60 |
| Polygonaceae |  |  |  | 0.99±1.72 |  | 5.02±4.35 | 2.23 | 0.21 |
| Aristolochiaceae |  |  | 2.61±4.53 | 6.96±7.95 | 2.81±4.87 | 0.82±1.43 | 0.74 | 0.56 |
| Scrophulariaceae |  |  |  | 1.04±1.8 |  |  |  |  |
| Campanulaceae |  |  |  |  | 2.69±3.21 |  |  |  |
| Rubiaceae | 2.63±4.56 | 1.69±2.92 | 4.58±7.92 |  | 0.83±1.44 |  | 0.33 | 0.80 |
| Caprifoliaceae |  |  |  |  |  | 3.47±4.47 |  |  |
| Dioscoreaceae | 1.75±3.04 |  | 2.61±4.53 |  |  |  | 0.05 | 0.84 |
