# Supplementary material for: Contrasting Responses of Rhizosphere Bacteria, Fungi and Arbuscular Mycorrhizal Fungi Along an Elevational Gradient in a Temperate Montane Forest of China
Source: Front Microbiol. 2020 Aug 20;11:2042. doi: 10.3389/fmicb.2020.02042 (PMC7469537; doi:10.3389/fmicb.2020.02042)
Supplement: Supplementary file 2 [file Table_2.docx]

**Table S2.** Relative abundances (average values and standard error) of bacterial composition in rhizospheric soil across taxonomical classification (Phyla, Class, and Order) along the elevation gradient. Different letters indicate significant differences (ANOVA, P < 0.05, Tukey’s HSD post-hoc analysis) among different elevation.

| ***Phyla*** | ***Class*** | ***Order*** | **Elevation gradient (m)** | | | | | | **F_(5, 12)_** | ***p*** |
| --- | --- | --- | --- | --- | --- | --- | --- | --- | --- | --- |
|  |  |  | **1308** | **1603** | **1915** | **2292** | **2405** | **2600** |  |  |
| *Prot* |  |  | 44.58±0.49D | 48.02±0.87C | 52.25±0.52A | 50.44±0.65AB | 48.32±0.37BC | 46.88±1.09C | 14.38 | **<0.001** |
|  | *Alph* |  | 32.07±0.76B | 33.93±1.42AB | 36.97±0.60A | 35.97±1.00A | 31.76±0.45BA | 26.84±1.59D | 11.79 | **<0.001** |
|  |  | *Rhiz* | 18.39±0.95CD | 28.55±1.60A | 24.82±0.63B | 23.76±0.65B | 21.63±0.75BC | 17.34±1.69D | 13.66 | **<0.001** |
|  |  | *Rhod* | 7.76±0.45A | 5.91±0.09C | 4.22±0.07D | 6.71±0.16B | 6.70±0.31B | 6.77±0.06B | 24.88 | **<0.001** |
|  |  | *Sphi* | 3.41±0.50A | 2.81±0.05A | 1.97±0.77B | 3.00±0.04A | 2.02±0.02B | 1.81±0.07B | 9.67 | **0.001** |
|  |  | *Caul* | 2.24±0.05A | 1.45±0.06B | 0.77±0.05D | 1.30±0.07C | 1.17±0.03C | 0.77±0.01D | 120.52 | **≤0.001** |
|  | *Beta* |  | 2.73±0.12E | 6.10±0.26BC | 4.93±0.32D | 5.91±0.08C | 6.88±0.11B | 8.68±0.43A | 60.70 | **≤0.001** |
|  |  | *Burk* | 1.72±0.05C | 2.31±0.16A | 1.65±0.02C | 1.88±0.10BC | 1.90±0.04BC | 2.15±0.11AB | 7.32 | **0.002** |
|  |  | *SC-I-84* | 0.88±0.03E | 1.40±0.16CD | 1.27±0.03D | 1.62±0.04C | 2.45±0.03B | 3.55±0.13A | 120.36 | **<0.001** |
|  |  | *Nitr* | 1.54±0.09B | 1.79±0.09B | 1.65±0.32B | 1.88±0.04B | 2.04±0.06AB | 2.49±0.19A | 4.34 | **0.017** |
|  | *Delt* |  | 3.69±0.05D | 3.71±0.26D | 5.34±0.05B | 4.03±0.03D | 4.80±0.17C | 7.07±0.09A | 94.16 | **<0.001** |
|  |  | *Desu* | 1.32±0.01E | 1.64±0.15D | 2.89±0.05B | 1.94±0.79C | 2.15±0.14C | 3.36±0.05A | 65.90 | **<0.001** |
|  |  | *Myxo* | 1.89±0.07A | 1.49±0.11BC | 1.73±0.04AB | 1.38±0.10C | 1.67±0.06AB | 1.49±0.06BC | 5.96 | **0.005** |
|  | *Gamm* |  | 5.09±0.13A | 4.26±0.22B | 4.33±0.12B | 3.52±0.27CD | 3.87±0.09BC | 3.29±0.08D | 15.02 | **<0.001** |
|  |  | *Xant* | 4.44±0.10A | 3.53±0.16BC | 3.20±0.03CD | 2.70±0.23E | 3.91±0.11B | 2.96±0.07DE | 22.34 | **<0.001** |
| *Acid* |  |  | 29.61±0.40A | 26.61±0.61B | 25.20±0.99BC | 24.39±0.76BC | 23.81±0.34CD | 21.89±0.86D | 14.23 | **<0.001** |
|  | *Subg6* |  | 5.38±0.15D | 7.28±0.14C | 7.89±0.11B | 7.64±0.10BC | 10.35±0.28A | 9.90±0.26A | 95.48 | **<0.001** |
|  | *Acid* |  | 13.83±0.11A | 7.66±0.17B | 2.93±0.24D | 4.23±0.26C | 3.39±0.06D | 2.32±0.02E | 685.93 | **<0.001** |
|  |  | *Acidob* | 3.83±0.11AB | 2.66±0.17DE | 2.93±0.24CD | 4.23±0.26A | 3.39±0.06BC | 2.32±0.02E | 18.56 | **<0.001** |
|  | *Soli* |  | 3.93±0.15A | 3.26±0.16B | 2.82±0.05B | 4.34±0.12A | 3.79±0.17A | 3.88±0.28A | 10.31 | **0.001** |
|  |  | *Solib* | 3.93±0.15A | 3.26±0.16B | 2.82±0.05B | 4.34±0.12A | 3.79±0.17A | 3.88±0.28A | 10.31 | **0.001** |
|  | *Subg2* |  | 2.77±0.21B | 2.82±0.31B | 1.31±0.07CD | 3.78±0.32A | 1.89±0.12C | 1.15±0.04D | 23.65 | **<0.001** |
|  | *Blas* |  | 1.53±0.15D | 2.33±0.30C | 3.30±0.11B | 2.48±0.42C | 2.83±0.11BC | 4.86±0.17A | 46.04 | **<0.001** |
|  |  | *Blas* | 1.53±0.15D | 2.33±0.30C | 3.30±0.11B | 2.48±0.42C | 2.83±0.11BC | 4.86±0.17A | 46.04 | **<0.001** |
|  | *Holo* |  | 1.18±0.08C | 0.94±0.06D | 1.94±0.03A | 0.85±0.06D | 1.33±0.00C | 1.67±0.14B | 30.58 | **<0.001** |
|  |  | *Subg7* | 0.81±0.03D | 0.66±0.06D | 1.64±0.07A | 0.61±0.04D | 1.08±0.01C | 1.37±0.12B | 38.59 | **<0.001** |
|  | *Subg17* |  | 0.69±0.03E | 1.22±0.06C | 1.54±0.11B | 0.61±0.03E | 1.04±0.02D | 1.88±0.02A | 82.730 | **<0.001** |
| *Acti* |  |  | 10.68±0.52A | 10.31±0.32A | 9.35±0.13B | 7.11±0.05C | 6.74±0.49C | 5.80±0.25D | 47.70 | **<0.001** |
|  | *Acti* |  | 5.80±0.40A | 5.30±0.35A | 4.02±0.17B | 2.83±0.22C | 2.39±0.01CD | 1.72±0.31D | 41.67 | **<0.001** |
|  | *Ther* |  | 2.56±0.06A | 2.45±0.13A | 1.76±0.11B | 1.77±0.13B | 1.98±0.02B | 1.74±0.11B | 15.45 | **<0.001** |
|  |  | *Gaie* | 1.56±0.05AB | 1.53±0.08AB | 1.67±0.09A | 1.26±0.09C | 1.36±0.03BC | 1.36±0.01BC | 5.412 | **0.008** |
|  |  | *Solir* | 1.00±0.04AB | 0.92±0.05B | 1.09±0.02A | 0.51±0.06CD | 0.62±0.03C | 0.38±0.06D | 40.31 | **<0.001** |
|  | *Acid* |  | 2.14±0.07CD | 2.33±0.03BC | 3.00±0.05A | 2.37±0.09B | 2.12±0.04CD | 2.06±0.09D | 28.01 | **<0.001** |
|  |  | *Acidi* | 2.14±0.07CD | 2.33±0.03BC | 3.00±0.05A | 2.37±0.09B | 2.12±0.04CD | 2.06±0.09D | 28.01 | **<0.001** |
| *Nitr* |  |  | 1.57±0.03E | 2.06±0.16DE | 2.64±0.03CD | 2.81±0.33C | 3.59±0.07B | 5.31±0.33A | 41.59 | **<0.001** |
|  | *Nitr* |  | 1.57±0.03E | 2.06±0.16DE | 2.64±0.03CD | 2.81±0.33C | 3.59±0.07B | 5.31±0.33A | 41.59 | **<0.001** |
|  |  | *Nitr* | 1.57±0.03E | 2.06±0.16DE | 2.64±0.03CD | 2.81±0.33C | 3.59±0.07B | 5.31±0.33A | 41.59 | **<0.001** |
| *Chlo* |  |  | 3.89±0.10D | 5.12±0.18C | 3.92±0.19D | 5.11±0.19C | 5.76±0.04B | 6.70±0.08A | 58.68 | **<0.001** |
|  | *KD4* |  | 1.88±0.08D | 2.67±0.16BC | 1.18±0.08E | 2.59±0.18C | 3.02±0.05AB | 3.23±0.10A | 41.62 | **<0.001** |
|  | *Anae* |  | 0.89±0.03BC | 1.05±0.04B | 0.88±0.08BC | 0.92±0.06BC | 0.80±0.29C | 1.55±0.62A | 24.41 | **<0.001** |
|  |  | *Anae* | 0.89±0.03BC | 1.05±0.04B | 0.88±0.08BC | 0.92±0.06BC | 0.80±0.29C | 1.55±0.62A | 24.41 | **<0.001** |
| *Gemm* |  |  | 3.17±0.19AB | 2.39±0.08C | 3.42±0.06A | 2.86±0.09B | 2.84±0.20B | 2.27±0.16C | 9.87 | **0.001** |
|  | *Gemm* |  | 3.17±0.19AB | 2.39±0.08C | 3.42±0.06A | 2.86±0.09B | 2.84±0.20B | 2.27±0.16C | 9.87 | **0.001** |
|  |  | *Gemm* | 3.17±0.19AB | 2.39±0.08C | 3.42±0.06A | 2.86±0.09B | 2.84±0.20B | 2.27±0.16C | 9.87 | **0.001** |
| *Plan* |  |  | 2.48±0.27B | 2.17±0.39BC | 1.57±0.13C | 3.40±0.08A | 2.05±0.02BC | 2.21±0.41BC | 5.34 | **0.008** |
|  | *Plan* |  | 2.08±0.23B | 1.78±0.32B | 1.09±0.08C | 3.05±0.80A | 1.50±0.20BC | 1.58±0.29BC | 10.81 | **<0.001** |
|  |  | *Plan* | 2.08±0.23B | 1.78±0.32B | 1.09±0.08C | 3.05±0.80A | 1.50±0.20BC | 1.58±0.29BC | 10.81 | **<0.001** |
| *Bact* |  |  | 2.06±0.12B | 1.73±0.15B | 1.99±0.07B | 2.20±0.22B | 3.07±0.15A | 2.06±0.15B | 9.19 | **0.001** |
|  | *Sphi* |  | 1.69±0.11B | 1.12±0.13C | 1.52±00.07B | 1.87±0.12B | 2.68±0.24A | 1.65±0.26B | 17.52 | **<0.001** |
|  |  | *Sphi* | 1.69±0.11B | 1.12±0.13C | 1.52±00.07B | 1.87±0.12B | 2.68±0.24A | 1.65±0.26B | 17.52 | **<0.001** |

**Phyla level:** *Proteobacteria (Prot), Acidobacteria (Acid), Actinobacteria (Acti), Nitrospirae (Nitr), Chloroflexi (Chlo), Gemmatimonadetes (Gemm), Planctomycetes (Plan), Bacteroidetes (Bact).*

**Class level**: *Alphaproteobacteria (Alph), Betaproteobacteria (Beta), Deltaproteobacteria (Dela), Gammaproteobacteria (Gamm), Subgroup_6 (Subg6), Solibacteres (Soli),* *Subgroup_2 (Subg2), Blastocatellia (Blas), Blastocatellia (Blas), Subgroup_17(Subg17), Actinobacteria (Acti), Thermoleophilia (Ther), Acidimicrobiia (Acid), Nitrospira (Nitr), KD4-96(KD4), Anaerolineae (Anae), Gemmatimonadetes (Gemm), Planctomycetacia (Plan), Sphingobacteriia (Sphi).*

***Order Level****: Rhizobiales (Rhiz), Rhodospirillales(Phod), Sphingomonadales (Sphi),* *Caulobacterales(Caul), Burkholderiales (Burk), SC-I-84(SC-I-84), Nitrospirales (Nitr), Desulfurellales (Desu), Myxococcales (Myxo), Xanthomonadales (Xant), Acidobacteriales (Adidob), Solibacterales (Solib), Blastocatellales (Blas), Subgroup_7(Subg), Gaiellales (Gaie), Solirubrobacterales (Solir), Acidimicrobiales (Acidi), Nitrospirales (Nitr), Anaerolineales (Anae), Gemmatimonadales (Gemm), Planctomycetales(Plan),* *Sphingobacteriales(Sphi).*
