# Supplementary material for: Contrasting Responses of Rhizosphere Bacteria, Fungi and Arbuscular Mycorrhizal Fungi Along an Elevational Gradient in a Temperate Montane Forest of China
Source: Front Microbiol. 2020 Aug 20;11:2042. doi: 10.3389/fmicb.2020.02042 (PMC7469537; doi:10.3389/fmicb.2020.02042)
Supplement: Supplementary file 3 [file Table_3.docx]

**Table S3.** Relative abundances (average values and standard error) of fungal composition in rhizospheric soil across taxonomical classification (Phyla, Class, and Order) along the elevation gradient. Different letters indicate significant differences (ANOVA, P < 0.05, Tukey’s HSD post-hoc analysis) among different elevation.

| ***Phyla*** | ***Class*** | ***Order*** | **Elevation gradient (m)** | | | | | | **F_(5, 12)_** | ***p*** |
| --- | --- | --- | --- | --- | --- | --- | --- | --- | --- | --- |
|  |  |  | **1308** | **1603** | **1915** | **2292** | **2405** | **2600** |  |  |
| *Basi* |  |  | 80.82±0.65AB | 82.05±0.68A | 76.90±0.52BC | 76.9±0.23BC | 75.07±1.89C | 64.08±2.09D | 26.86 | **<0.001** |
|  | *Agar* |  | 80.09±0.69A | 81.44±0.70A | 74.63±0.54B | 75.75±0.17B | 74.30±1.70B | 62.79±2.26C | 28.08 | **<0.001** |
|  |  | *Agar* | 36.22±0.89B | 45.44±3.46A | 34.12±1.15B | 46.83±3.38A | 45.40±4.28A | 23.35±2.24C | 10.31 | **0.001** |
|  |  | *Russ* | 18.61±0.57A | 6.78±1.37BC | 14.78±3.33A | 8.76±1.86B | 3.63±0.42BC | 3.08±0.28C | 13.71 | **<0.001** |
|  |  | *Thel* | 6.63±0.16B | 4.22±0.75BC | 6.97±1.86B | 7.62±1.25AB | 2.96±0.27C | 10.76±1.37A | 5.95 | **0.005** |
|  |  | *Seba* | 2.38±0.06C | 1.68±0.25C | 2.76±0.47C | 2.61±0.16C | 4.31±0.41B | 17.6±0.91A | 173.70 | **<0.001** |
|  |  | *Cant* | 0.27±0.02A | 6.67±1.08A | 6.61±4.52A | 0.75±0.05A | 0.78±0.03A | 5.01±1.47A | 2.38 | **0.102** |
|  |  | *Bole* | 3.96±0.51B | 8.26±0.81A | 3.59±0.67BC | 0.98±0.02DE | 2.26±0.38CD | 0.23±0.04E | 31.61 | **<0.001** |
|  |  | *Trec* | 0.73±0.12B | 0.53±0.08B | 0.61±0.11B | 0.69±0.02B | 10.08±4.90A | 0.66±0.02B | 3.71 | **0.029** |
|  | *Trem* |  | 0.50±0.02B | 0.50±0.11B | 2.11±0.38A | 0.32±0.12B | 0.67±0.19B | 0.96±0.24B | 10.27 | **0.001** |
|  |  | *Trem* | 0.49±0.02B | 0.48±0.10B | 1.95±0.42A | 0.32±0.02B | 0.67±0.19B | 0.96±0.19B | 8.28 | **0.001** |
| *Asco* |  |  | 16.89±0.63C | 12.36±0.54D | 18.25±0.39C | 17.84±0.61C | 22.45±1.90B | 28.53±2.35A | 17.15 | **<0.001** |
|  | *Leot* |  | 1.93±0.12B | 4.07±0.81B | 5.26±1.83B | 3.59±0.03B | 17.22±1.77A | 14.78±0.46A | 34.11 | **<0.001** |
|  |  | *Helo* | 0.92±0.09A | 1.46±0.19A | 3.20±1.64A | 1.77±0.08A | 3.08±0.37A | 2.17±0.23A | 1.67 | **0.216** |
|  |  | *Leot* | 0.39±0.09B | 0.32±0.08B | 0.27±0.14B | 1.56±0.10B | 13.66±1.41A | 1.71±0.25B | 79.32 | **<0.001** |
|  |  | *Ince* | 0.43±0.05CD | 0.86±0.03A | 0.56±0.11BC | 0.17±0.01D | 0.36±0.01E | 0.61±0.03B | 19.84 | **<0.001** |
|  | *Sord* |  | 7.94±0.73A | 3.07±0.15BC | 5.30±2.07AB | 5.70±0.09AB | 0.75±0.07C | 3.23±1.59BC | 5.13 | **0.010** |
|  |  | *Sord* | 5.95±0.75A | 1.03±0.66BC | 1.94±0.62B | 0.06±0.00C | 0.11±0.02C | 0.29±0.09C | 32.13 | **<0.001** |
|  |  | *Hypo* | 1.79±0.04AB | 1.72±0.12ABC | 2.46±0.95A | 0.31±0.05C | 0.29±0.03BC | 1.44±0.41ABC | 3.88 | **0.025** |
|  | *Doth* |  | 0.99±0.04BC | 1.35±0.17BC | 2.29±0.39A | 0.77±0.05C | 1.50±0.08B | 2.19±0.27A | 8.50 | **0.001** |
|  |  | *Myri* | 0.52±0.01C | 1.00±0.13B | 0.89±0.07B | 0.53±0.05C | 1.32±0.08A | 1.44±0.07A | 24.97 | **<0.001** |
|  | *Euro* |  | 3.94±0.58A | 2.70±0.10A | 2.86±0.67A | 0.65±0.04B | 0.60±0.16B | 1.03±0.28B | 12.87 | **<0.001** |
|  |  | *Chae* | 0.99±0.03AB | 1.51±0.05AB | 2.02±0.84A | 0.58±0.40B | 0.39±0.02B | 0.28±0.14B | 3.09 | **0.051** |
|  |  | *Euro* | 2.94±0.61A | 1.19±0.05B | 0.82±0.17BC | 0.06±0.01C | 0.21±0.14C | 0.20±0.10C | 16.49 | **<0.001** |
|  | *Pezi* |  | 0.27±0.02ABC | 0.32±0.04AB | 0.46±0.03A | 0.09±0.03C | 0.18±0.02BC | 0.45±0.13A | 5.92 | **0.006** |
|  |  | *Pezi* | 0.27±0.02ABC | 0.32±0.04AB | 0.46±0.03A | 0.09±0.03C | 0.18±0.02BC | 0.45±0.13A | 5.92 | **0.006** |
| *Zygo* |  |  | 1.14±0.02C | 4.30±0.58AB | 3.74±0.53B | 4.87±0.38AB | 1.79±0.03C | 5.81±0.93A | 14.78 | **<0.001** |
|  | *Ince* |  | 1.14±0.02C | 4.30±0.58AB | 3.74±0.53B | 1.20±0.52C | 1.79±0.03C | 5.81±0.93A | 12.04 | **<0.001** |
|  |  | *Mort* | 1.10±0.02C | 4.21±0.57B | 3.66±0.54B | 1.17±0.50C | 1.77±0.04C | 5.77±0.93A | 14.73 | **<0.001** |

**Phyla level:** *Basidiomycota (Basi), Ascomycota (Asco), Zygomycota (Zygo).*

**Class level**: *Agaricomycetes (Agar), Tremellomycetes (Trem), Leotiomycetes (Leot), Sordariomycetes (Sord), Dothideomycetes (Doth), Eurotiomycetes (Euro), Pezizomycetes (Pezi), Incertaesedis (Ince).*

***Order Level****: Agaricales (Agar), Russulales (Russ), Thelephorales (Thel), Sebacinales (Seba), Cantharellales (Cant), Boletales (Bole)，Trechisporales (Trec), Tremellales (Trem),* *Helotiales(Helo),* *Leotiales (Leot), Incertae sedis (Ince), Sordariales (Sord), Hypocreales (Hypo),* *Myriangiales(Myri), Chaetothyriales(Chae), Eurotiales (Euro), Pezizales (Pezi),* *Mortierellales (Mort).*
