# Supplementary material for: Contrasting Responses of Rhizosphere Bacteria, Fungi and Arbuscular Mycorrhizal Fungi Along an Elevational Gradient in a Temperate Montane Forest of China
Source: Front Microbiol. 2020 Aug 20;11:2042. doi: 10.3389/fmicb.2020.02042 (PMC7469537; doi:10.3389/fmicb.2020.02042)
Supplement: Supplementary file 4 [file Table_4.docx]

**Table S4.** Relative abundances (average values and standard error) of AMF composition in rhizospheric soil across taxonomical classification (Phyla, Class, and Order) along an elevation gradient. Different letters indicate significant differences (ANOVA, P < 0.05, Tukey’s HSD post-hoc analysis) among different elevation

| ***Phyla*** | ***Class*** | ***Order*** | **Elevation gradient (m)** | | | | | | **F_(5, 12)_** | ***p*** |
| --- | --- | --- | --- | --- | --- | --- | --- | --- | --- | --- |
|  |  |  | **1308** | **1603** | **1915** | **2292** | **2405** | **2600** |  |  |
| *Glom* |  |  | 97.36±0.45BC | 95.93±0.6C | 96.29±1.07C | 99.45±0.19A | 98.47±0.39AB | 98.68±0.58AB | 15.99 | **<0.001** |
|  | *Para* |  | 84.82±2.5AB | 74.33±2.52B | 50.64±5.48C | 92.32±1.96A | 80.6±2.81B | 82.18±3.24B | 57.56 | **<0.001** |
|  |  | *Para* | 84.82±2.5AB | 74.33±2.52B | 50.64±5.48C | 92.32±1.96A | 80.6±2.81B | 82.18±3.24B | 57.56 | **<0.001** |
|  | *Glom* |  | 10.72±2.34C | 19.95±1.87B | 43.09±5.09A | 3.57±1.39D | 14.7±2.69BC | 15.13±2.51BC | 65.32 | **<0.001** |
|  |  | *Glom* | 10.6±2.35C | 19.51±1.86B | 42.23±5.31A | 3.45±1.38D | 13.9±2.41BC | 13.95±2.64BC | 61.42 | **<0.001** |

**Phyla level:** *Glomeromycota (Glom).*

**Class level**: *Paraglomeromycetes (Para), Glomeromycetes (Glom).*

***Order Level****: Paraglomerales (Para), Glomerales (Glom).*
